# Supplementary material for: High resolution respirometry of isolated mitochondria from adult Octopus maya (Class: Cephalopoda) systemic heart
Source: PLoS One. 2022 Aug 29;17(8):e0273554. doi: 10.1371/journal.pone.0273554 (PMC9423623; doi:10.1371/journal.pone.0273554)
Supplement: S1 File — (PDF) [file pone.0273554.s001.pdf]

High resolution respirometry of isolated mitochondria from adult *Octopus maya* (Class: Cephalopoda) systemic heart V.2

Version 2

Ana Karen Meza-Buendía[1], Omar Emiliano Aparicio-Trejo[2], Fernando Díaz-Herrera[1], Claudia Caamal-Monsreal[3,4], José Pedraza-Chaverri[5], Carolina Álvarez-Delgado[6], Kurt Paschke[7,8], Carlos Rosas[3,4]

[1]Laboratorio de Ecofisiología de Organismos Acuáticos, Departamento de Biotecnología Marina, Centro de Investigación Científica y de Educación Superior de Ensenada (CICESE), Ensenada, Baja California, México;

[2]Departamento de Fisiopatología Cardio-Renal, Instituto Nacional de Cardiología "Ignacio Chávez", Mexico City 14080, Mexico;

[3]Unidad Multidisciplinaria de Docencia e Investigación, Facultad de Ciencias, Universidad Nacional Autónoma de México, Sisal, Mexico;

[4]Laboratorio de Resilencia Costera (LANRESC, CONACYT, Sisal, Mexico;

[5]Laboratorio F-315, Departamento de Biología, Facultad de Química, Universidad Nacional Autónoma de México, 04510, Ciudad de México, Mexico;

[6]Departamento de Innovación Biomédica, Centro de Investigación Científica y de Educación Superior de Ensenada (CICESE), Carretera Tijuana-Ensenada 3918, Zona Playitas, Baja California, Mexico;

[7]Instituto de Acuicultura, Universidad Austral de Chile, Puerto Montt, Chile;

[8]Centro FONDAP de Investigación de AltasLatitudes (IDEAL), Punta Arenas, Chile

dx.doi.org/10.17504/protocols.io.yxmvmn3z6g3p/v2

Ana Karen Meza-Buendia

ABSTRACT

Mitochondrial respirometry is key to understand how environmental factors model energetic cellular process. In the case of ectotherms, thermal tolerance has been hypothesized to be intimately linked with mitochondria capability to produce enough adenosine triphosphate (ATP) to respond to the energetic demands of animals in high temperatures. Recent studies made in *Octopus maya* proposed the hypothesis postulating that high temperatures could restrain female reproduction due to the limited capacity of the animals' heart to sustain oxygen flow to the body, affecting in this manner energy production in the rest of the organs, including the ovarium. Until now, no reports have shown temperature effects and other environmental variables on cephalopod mitochondria activity because of the lack of a method to evaluate mitochondrial respiratory parameters on those groups of species. In this sense and for the first time, this study developed a method to obtain mitochondrial respirometry data of adult *Octopus maya*'s heart. This protocol illustrates a step-by-step procedure to get high yield and functional mitochondria of cephalopod heart and procedure for determining the corresponding respiratory parameters. The isolation procedures described here require two hours, demonstrating that confident and replicable results can be obtained with this method.

ATTACHMENTS

High resolution respirometry of isolated mitochondria from adult Octopus maya (Class Cephalopoda) systemic heart.docx

DOI

dx.doi.org/10.17504/protocols.io.yxmvmn3z6g3p/v2

PROTOCOL CITATION

Ana Karen Meza-Buendía, Omar Emiliano Aparicio-Trejo, Fernando Díaz-Herrera, Claudia Caamal-Monsreal, José Pedraza-Chaverri, Carolina Álvarez-Delgado, Kurt Paschke, Carlos Rosas . High resolution respirometry of isolated mitochondria from adult Octopus maya (Class: Cephalopoda) systemic heart. **protocols.io**
https://dx.doi.org/10.17504/protocols.io.yxmvmn3z6g3p/v2
Version created by Ana Karen Meza-Buendia

KEYWORDS

Mitochondria, Heart, High resolution respiration, Octopus maya, Respiratory parameters, Mitochondrial isolation

LICENSE

This is an open access protocol distributed under the terms of the Creative Commons Attribution License, which permits unrestricted use, distribution, and reproduction in any medium, provided the original author and source are credited

CREATED

Jul 12, 2022

LAST MODIFIED

Jul 12, 2022

PROTOCOL INTEGER ID

66497

MATERIALS TEXT

Chemicals for Mitochondrial Isolation Buffer and Mitochondrial Respiratory Buffer

1. Sucrose (Sigma, cat. no. S 9378)
2. Potassium chloride (KCl, Sigma, cat. no. P 5405)
3. Ethylene-bis (oxyethylenenitrilo) tetraacetic acid (EGTA, Sigma, cat. no. E3889)
4. 4-(2-Hydroxyethyl) piperazine-1-ethanesulfonic acid (HEPES, Sigma, cat. no. H 4034)
5. Bovine serum albumin (BSA) fatty acid free (Sigma, cat. no. A 7030)
6. Magnesium chloride ($MgCl_2$, Sigma, cat. no. 208337)
7. Lactobionic acid (Sigma, cat. no. 15316)
8. Taurine (Sigma, cat. no. T0625)

9. Potassium phosphate monobasic (KH_2PO_4, Sigma, cat. no. P5379)

Chemicals for respiratory substrates and inhibitors

1. L-proline (Sigma, cat. no. P 0380)
2. Adenosine 5 ’diphosphate potassium salt (ADP; Sigma, cat. no. A 5285)
3. Oligomycin from *Streptomyces diastatochromogenes*(Sigma, cat. no O 4876)
4. Antimycin A (from *Streptomyces* sp., Sigma, cat. no A 8674)
5. Rotenone ($C_{23}H_{22}O_6$, Sigma, cat. no. 45656)

Other chemicals

1. Ethanol (70 %)
2. Bi-distilled water
3. 5 M Potassium hydroxide (KOH, Sigma, cat. no 484016)

Preparation of mitochondrial isolation buffers

Mitochondrial Isolation Buffer A: Mix all the reagents in Table 1 except BSA- Fatty Acid-Free. Once dissolved, the pH of the buffer is adjusted to 7.4. The pH is adjusted with 5 M KOH. If not used immediately, make aliquots in 50 mL falcon® tubes and stored at -80°C for up to six months.
If used on the same day of preparation (after pH adjustment), take an aliquot (50 mL), and add the corresponding amount of BSA (1 g/L). Store the other aliquots at -80°C.

Mitochondrial isolation buffer B: Mix all the reagents from Table 1 without adding the BSA- Fatty Acid-Free. Adjust pH to 7.4 with 5M KOH. If not used immediately prepare aliquots in 50 mL falcon® tubes and store at -80°C for up to six months.If used on the same day as preparation, it can be used after pH adjustment.

Table 1. Modified isolation buffer from Mommsen and Hochachka (1981)

A	B	C	D	E	F
**Component**	**MW g/mol**	**Molarity (mM)**	**Osmoles**	**Osmolarity**	**Amount for 200 mL final volume [g]**
Sucrose	342.3	500	1	500	34.23
KCl	74.55	150	2	300	2.23
EGTA	380.35	2	1	2	0.15
HEPES	238.30	25	1	25	1.19
BSA		1g/L			0.200
				826 mOsmoles	

Experiment day: Both mitochondrial isolation buffers are thawed at room temperature or in a 36°C water bath. Once the *mitochondrial isolation buffer A* isthawed, add the BSA- Fatty Acid-Free(Table 1). Dissolve 0.05 g for a 50 mL aliquot. *Mitochondrial isolation buffer B* once completely thawed can be used.
When both isolation buffers are used on the day of the experiment, they are kept cold. Once opened, they can be refrozen at -20°C and used within six days.

MiR05 mitochondrial respiratory buffer preparation based on Gnaigner *et al.* (2018)

Table 2. Mitochondrial respiration buffer MiR05 (Gnaiger *et al.* 2018)

A	B	C	D
**Component**	**MWMW g/mol**	**Final concentration [mM]**	**Amount for 250 mL final volume [g]**
EGTA	380.4	0.5	0.047
MgCl2	95.2	3	0.071
Lactobionic acid	358.3	60	5.375
Taurine	125.1	20	0.625
KH2PO4	136.1	10	0.340
HEPES	238.3	20	1.191
Sucrose	342.3	110	9.413
BSA		1g/L	0.250

1. Transfer all the reagents from Table 2 except the BSA-free fatty acids into a beaker.
2. Add 230 mL of double-distilled water.
3. Dissolve with magnetic stirring at 30 °C.
4. Add 3.75 mL of 5 M KOH at 30 °C and stir for 90 min.
5. Adjust pH 7.1 with 5 M KOH at 30 °C using a pH electrode. The pH adjustment can be slow (90 min).

pH must be stable for at least 5 min. Do not leave the pH electrode in the solution for the 90-min waiting time

6. Separate 50 mL aliquots in Falcon (Falcon Tubes Pvt Ltd) tubes and store at -80 °C (the MiR05 will have a 6-month shelf life).

Use on the day of experiment:

1. Thaw at room temperature or in a water bath at 36 ° C.
2. Once thawed add 0.05 g of BSA in 50 mL of MiR05.
3. Keep cold until use.

Preparation of substrates and inhibitors for high-resolution respirometry

The substrates and inhibitors used in this methodology are prepared according to the information available online from Oroboros Instrument. However, its preparations are described below, for more information consult https://wiki.oroboros.at/index.php/OROBOROS_INSTRUMENTS.

2 M Proline stock solution: Dissolve 1151.30 mg of Proline in 5 ml of bio-distilled water.Prepare 500 µl aliquots and store at -20 °C.
500 mM ADP stock solution: Dissolve 501.3 mg of ADP in 1.2 mL of bio-distilled water (ADP does not dissolve at this stage), neutralize with 5 M KOH (~ 450 µl), and check pH 7.

Adjust the final volume to 2 ml. Prepare 200 µl aliquots and store at -80 °C.
5 mM Oligomycin stock solution: Dissolve 4 mg of oligomycin in 1 ml of ethanol 70%. Prepare 200 µl aliquots and store at -20 °C.
1 mM Rotenone stock solution: Dissolve 0.39 mg of rotenone in 1 ml of ethanol 70%. Prepare 200µl aliquots and store at -20 ° C.
5 mM Antimycin A stock solution: Dissolve 5.4 mg of antimycin A in 2 mL ml of 70% ethanol solution. Prepare 200 µl aliquots and store at -20 °C.

Isolation of mitochondria from the systemic heart of adult octopus

1

BEFORE STARTING: Pre-chill glassware before starting the procedure.

Starve octopus overnight before the isolation experiment.

2 Sacrifice an adult *Octopus maya* specimen (about 1 kg) previously anesthetized with 3% alcohol and quickly remove the systemic heart from the mantle cavity.

CRITICAL STEP: To obtain mitochondria from the systemic heart of *O. maya*, a minimum of 0.5 g of tissue is used.

3 Place the systemic heart immediately on a Petri dish on ice and add 1 mL of *mitochondrial Isolation Buffer A* to rinse the organ.

4 Cut the systemic heart into pieces with scissors and mince into smaller pieces with a scalpel, which should be done while the Petri dish is on ice.

5 Transfer the cut pieces of the organ to a homogenization tube with 2 ml of cold mitochondrial isolation buffer A.

NOTE: Homogenization, as well as the following steps, must be carried out at 4 °C.

6 Homogenize the systemic heart using Potter-Elvehjem PTFE pestle and glass tube (Sigma-Aldrich P7859-1EA) homogenizer operated by a drill at 500 rpm. Three to four stocks are made to homogenize the previously minced tissue. Homogenization is done in a container with ice and the ice homogenization tube must not be removed.

CRITICAL STEP: The drill pistil must enter rotating to avoid forming bubbles and generating surface tension causing the isolated mitochondria to burst.

7 Transfer the homogenate by decantation to a pre-cooled 2-ml Eppendorf tube® and centrifuge at 392 rfc at 4 °C and kept on ice for 5 min. If the centrifuge is not nearby, keep the tube with the homogenate cold.

8 Transfer the supernatant obtained from the previous step to another pre-cooled 2-ml Eppendorf tube® with a micropipette and keep on ice.

CRITICAL STEP*:* Hold the Eppendorf tube ® by the top of the cap to avoid heating it and keep it on ice.

9 Centrifuge the transferred supernatant at 7,939 rfc for 15 min at 4 °C ('mitochondrial pellet formation').

10 Discard the supernantant by decantation and wash off the pellet. First add 1 ml of cold*mitochondrial isolation buffer B* and re-suspend the pellet gently with a soft bristle brush (natural bristles).

CRITICAL STEP: Decantation should be quick and avoid leaving the tube without ice as much as possible.
CRITICAL STEP: Resuspension of the pellet is performed on ice without lifting the Eppendorf tube ®.

11 Re-suspend the pellet, add 1 ml of cold *mitochondrial isolation buffer B*. Subsequently shake gently and quickly to homogenize and keep on ice.

12 Centrifuge at 7,938 rfc at 4 °C for 15 minutes.

13

Discard the supernatant by decantation and conserve the pellet.

CRITICAL STEP: Decantation should be quick and avoid leaving the tube without ice as much as possible.

14 Add 160 µL of cold mitochondrial isolation buffer to concentrate the sample and resuspend the pellet in the same way as in steps 10 and 11. Keep cold.

15 Measure mitochondrial concentration using the Bradford method (Bradford 1976). According to our own experimental results, mitochondrial suspensions from the systemic heart of *Octopus maya* adults contain aproximatly 14 mg protein/ml per 1 gram of minced tissue. Mitochondria are now ready to be used in experiments of respirometry; use the preparation within 1–4 h for better functional responses.

CRITICAL STEP: Keep the mitochondrial fraction on ice.

16 **NOTE:** A diagram of the summary steps of the isolation of mitochondria from *Octopus maya* is shown in Fig 1.

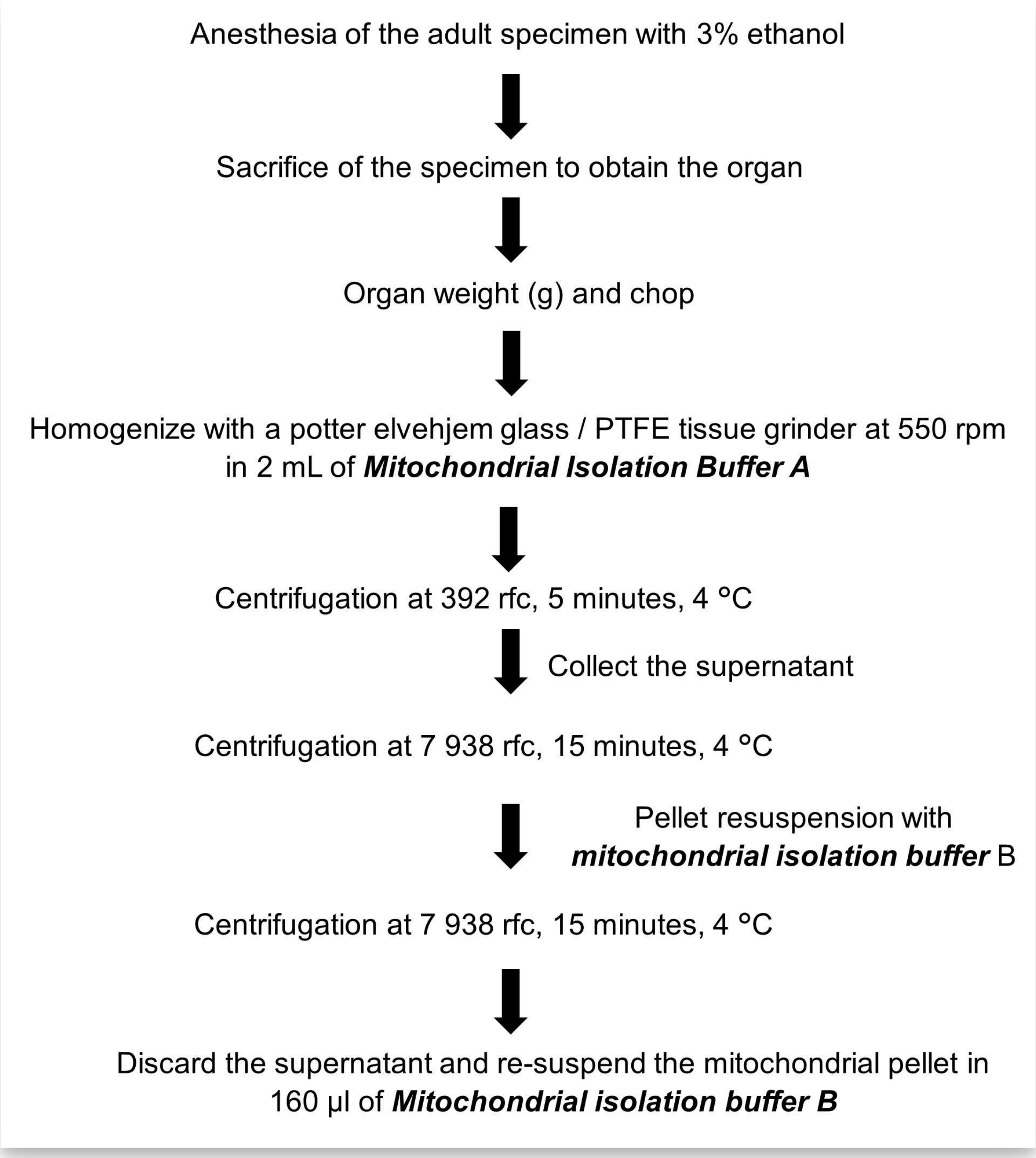

Fig 1. Mitochondrial isolation of a systemic heart from an adult *Octopus maya*.

Measuring mitochondrial respiration: High-resolution respirometry (HRR)

17

The following protocol is designed to be used in a commercially available HRR device, the Oxygraph™ O2k (Oroboros Instruments, Innsbruck, AT), which uses a polarographic oxygen sensor to detect oxygen (O_2) flux of ± 1 pmol $O_2 \cdot s^{-1} \cdot mL^{-1}$. To adapt the protocol to other commercial equipment, please see the manufacturer's specifications. The equipment should be turned on before the mitochondrial isolation starts, so it reaches the selected experimental working temperature (the data shown in this document were determined at temperature of 24 °C).

Equipment setup: Calibration of polarographic oxygen sensors

18 Add 2 mL of mitochondrial respiration buffer (MiR05) to the chamber (this protocol was developed using a 2 ml volume), and the O_2 sensors are calibrated.

NOTE: Mitochondrial Respiration Buffer MiR05 should be used instead of distilled water for calibration

19 Wait for an equilibrium with atmospheric oxygen and the required experimental temperature; the system reaches the steady basal consumption state of the system in operation, a point where the O_2 consumption rate is constant.

20 Start recording of oxygen consumption. Verify that the recording is stable and that no drifts are apparent.

Substrate/inhibitor titration (SUIT) analysis

21

This section provides a SUIT protocol for the analysis of oxidative phosphorylation (OXPHOS) in *Octopus maya* systemic heart mitochondria, being a tool for understanding the mitochondrial respiratory control of this species. See Table 1, to consult the concentrations of the substrates and inhibitors used in this protocol.

A	B	C
**Reagent**	**Action**	**Final concentration**
Proline	Amino acid substrate	5 mM
ADP	Substrate for the generation of ATP	1.25 mM
Antimycin	Complex III inhibitor	12.5 µM
Rotenone	Complex I inhibitor	2.5 µM
Oligomycin	ATP synthase inhibitor	2.5 µM

Table 1. Action and concentration of agents used for measuring mitochondrial respiration of isolated mitochondria from the systemic heart of *Octopus maya*.

22 Use an appropriate Hamilton microsyringe (Oroboros Instrument), add mitochondria (**Mtc**) to obtain a final concentration between 300-500 µg ml^{-1}. This step is followed by a rapid and transient decrease in oxygen content of the chamber followed by a slower decrease caused by respiration of the mitochondria, commonly referred to as Respiratory State 1.

NOTE: 600 to 1000 µg of total protein are recommended.

23 Use a Hamilton microsyringe (Oroboros Instruments), add Proline (**Pro**) to a final concentration of 5 mM.

CRITICAL STEP: The corresponding respiratory substrates must be immediately added to avoid mitochondrial membrane potential depolarization.
NOTE: The addition of proline starts proline pathway (entry in electron transport system direct into Q-junction) and the glutamate-anaplerotic pathway (stimulates CI-linked respiration). Proline is oxidized to 1-delta pyrroline 5 carboxylate by proline dehydrogenase of the inner mitochondrial membrane reducing FAD to FADH2, where 1-delta pyrroline 5 carboxylate is converted to glutamate by 1 pyrroline 5 carboxylate dehydrogenase. Additionally, FADH2 is oxidized to stimulate quinone reduction, activating Q-junction.

24 Observe a faster rate of oxygen consumption because of basal activity of the respiratory chain to counteract proton leakage from the inner mitochondrial membrane, which represents Respiratory State 2' (**S2'**).

25 Record for ~ 2 min.

26 Add 5 µl **ADP** (500 mM ADP stock solution) to obtain a final concentration of 1.25 mM. A faster oxygen consumption is observed and represents Respiratory State 3' (**S3'**), where ATP production is the principal contribution of oxygen consumption.

CRITICAL STEP: The rate of oxygen consumption should be faster than the rate of consumption observed when adding the substrate alone, indicating that well-coupled mitochondria have been obtained.

27 Record until the rate of oxygen consumption begins to drop.

28 Add 1 µl of **oligomycinA**(5 mM oligomycin stock solution) to obtain a final concentration of 2.5 µM and induce Respiratory State 4' (**S4'o**). With this procedure OXPHOS is inhibited by oligomycin and the rate of oxygen consumption begins to rapidly plateau (steady state).

29 Record for ~ 2 min.

30 Add 2.5 µM rotenone (**Rot**) plus 12.5 µM antimycin A (**Ant**) to obtain residual or non-mitochondrial respiration (**ROX**). Both compounds inhibit the electron transport system flux and induce a rapid decrease in oxygen consumption rate until it remains constant.

31 Record for ~5 min and then stop recording.

32 The Respiratory States **S3'** and **S4'o**, were corrected for the respiratory state **ROX** (residual non-mitochondrial respiration): S3= **S3'**-**ROX** and S4= **S4'o**-**ROX**. The respiratory control parameter (RC) was defined as S3**/**S4o**,**while respiration directly attributable to OXPHOS was defined as S3-S4o, which is the phosphorylation state parameter (*P*). See Fig 2.

NOTE: To correctly determine O_2 consumption rate in each Respiratory State, it is necessary to ensure that the steady-state is reached.
NOTE: To avoid hypoxia in the chambers, they must be reoxygenated (by chamber opening) if O_2 concentration falls below 20 µM.

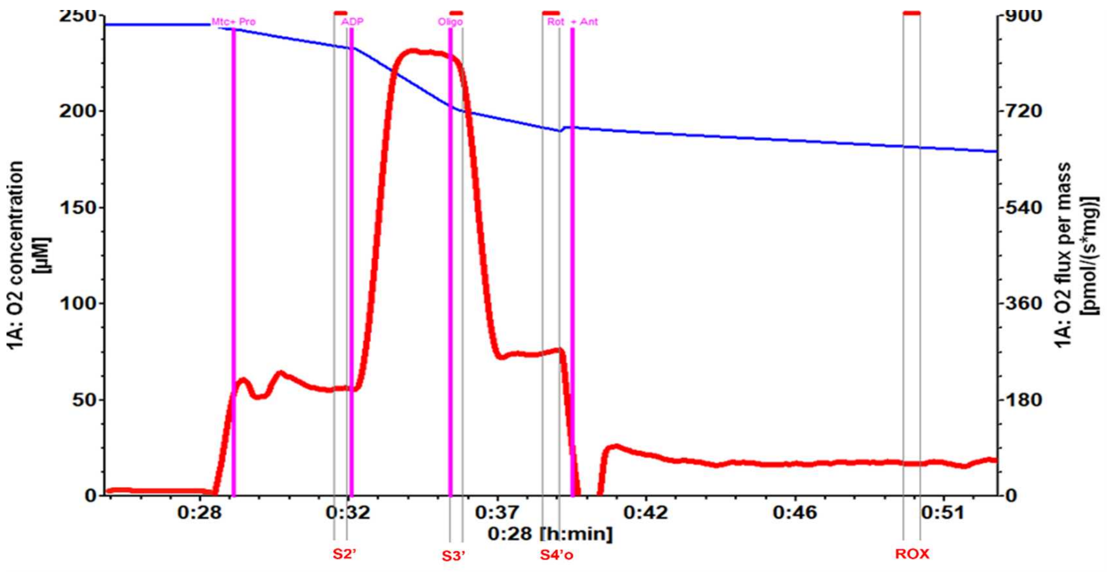

Fig 2. Schematic representations of the method used to determine the rate of oxygen consumption in each respiratory state (S2', S3', S4'o and ROX); the blue line corresponds to O_2 concentration (µM), while the red linecorresponds to oxygen consumption rate (pmol O_2 s^{-1} mg^{-1}). **Mtc**: mitochondria, **Pro**: proline, **ADP**: adenosine diphosphate, **Oligo**: oligomycin, **Rot**: rotenone and **Ant**: antimycin A. **S2'**=State 2';**S3'** = State 3'; **S4'o**= state 4 oligomycin-induced; **ROX**= residual non- mitochondrial respiration; Rot + Anyt = rotenone plus antimycin.
