# Supplementary figures and images for: High resolution respirometry of isolated mitochondria from adult Octopus maya (Class: Cephalopoda) systemic heart
Source: PLoS One. 2022 Aug 29;17(8):e0273554. doi: 10.1371/journal.pone.0273554 (PMC9423623; doi:10.1371/journal.pone.0273554)

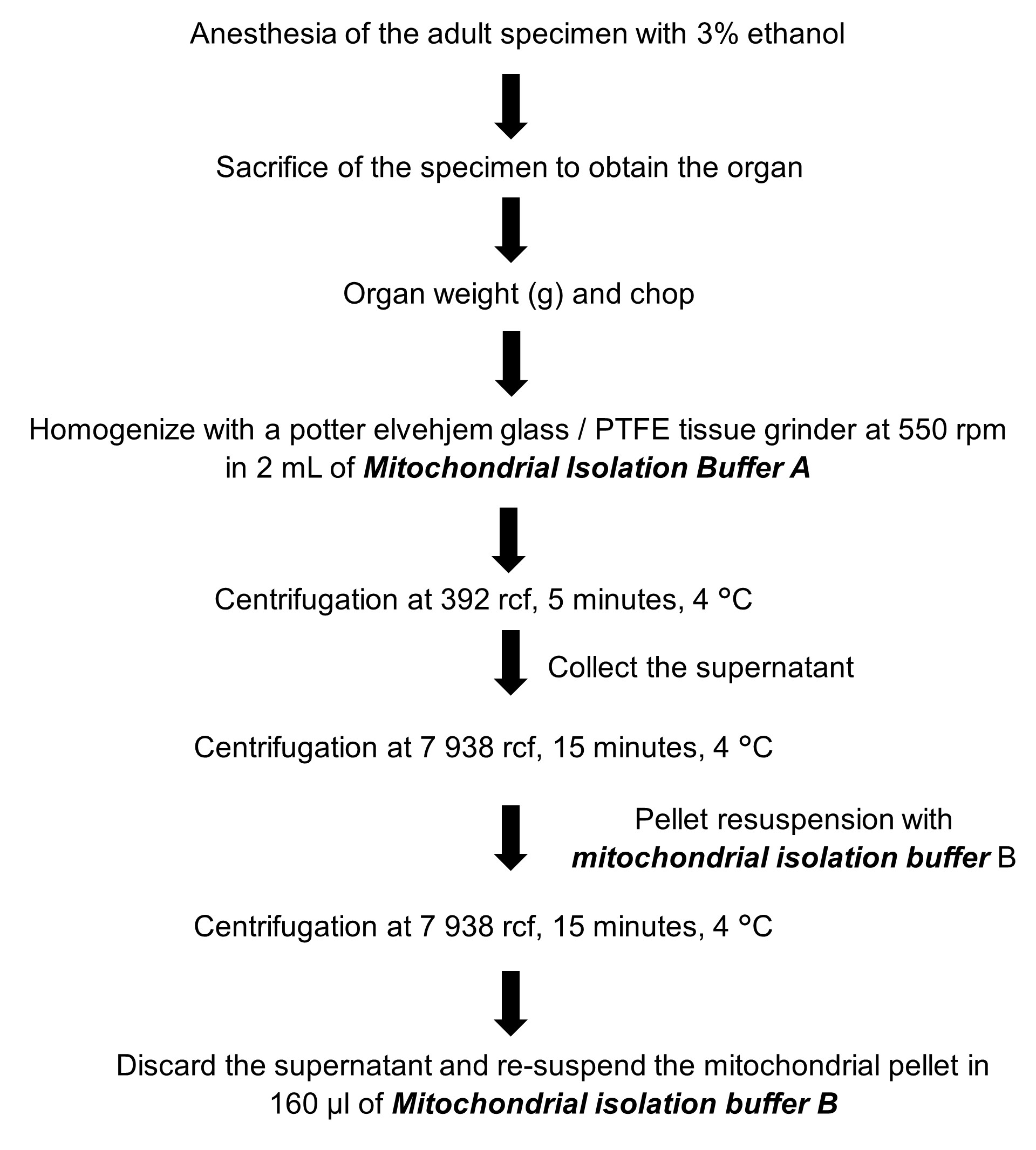

Supplement: S1 Fig — (TIF) [file pone.0273554.s002.tif]

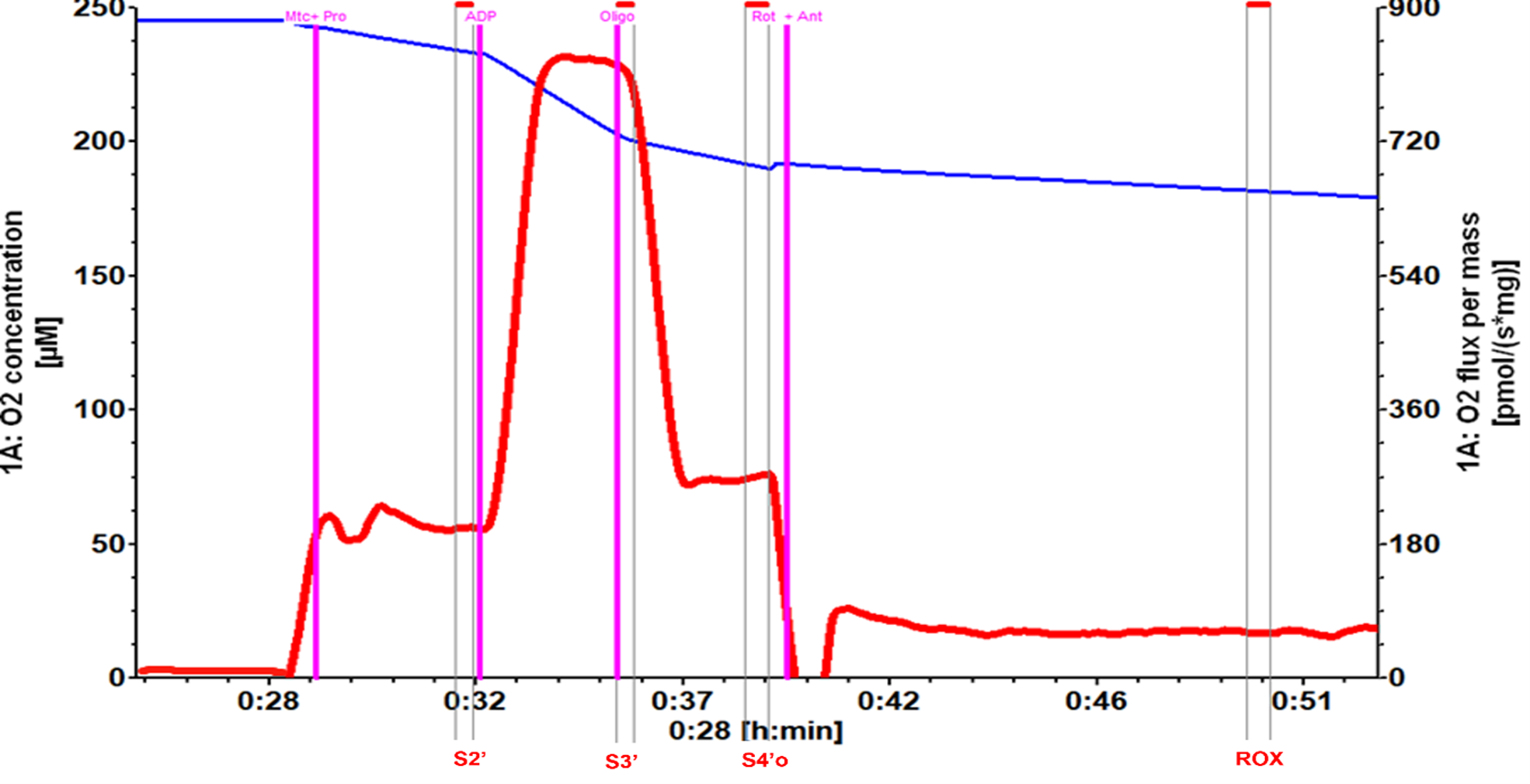

Supplement: S2 Fig — (TIF) [file pone.0273554.s003.tif]

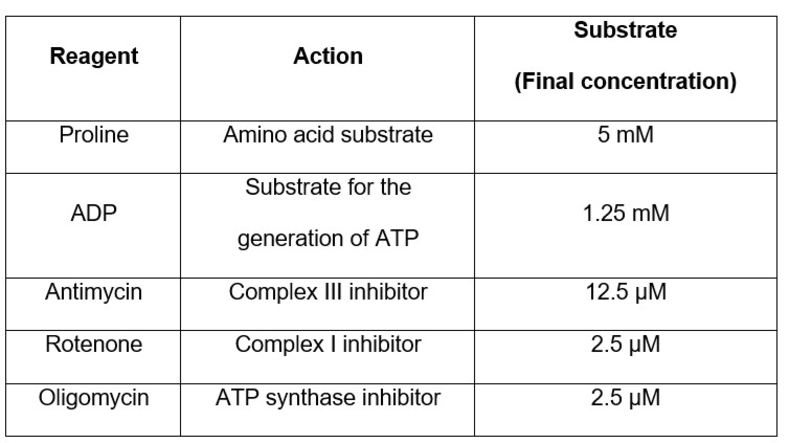

Supplement: S1 Table — (TIF) [file pone.0273554.s004.tif]

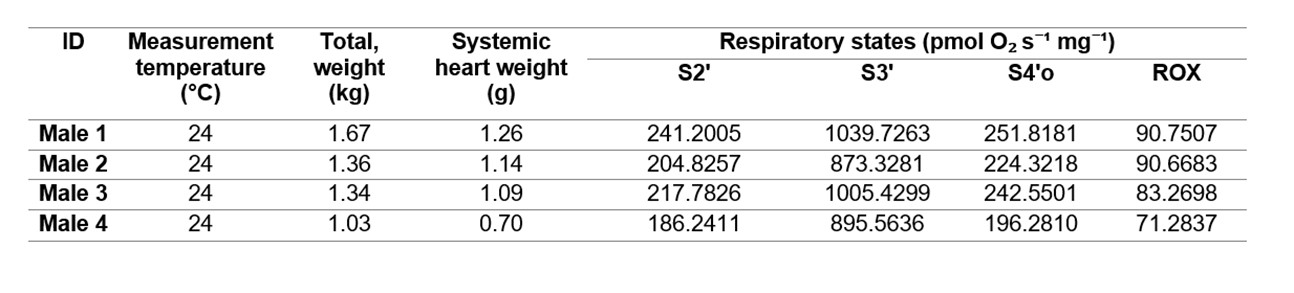

Supplement: S2 Table — (TIF) [file pone.0273554.s005.tif]
