# Supplementary material for: High resolution respirometry of isolated mitochondria from adult Octopus maya (Class: Cephalopoda) systemic heart
Source: PLoS One. 2022 Aug 29;17(8):e0273554. doi: 10.1371/journal.pone.0273554 (PMC9423623; doi:10.1371/journal.pone.0273554)
Supplement: Attachment — Submitted filename: PlosOne-D-22-06578.docx [file pone.0273554.s006.docx]

Carlos Rosas Vazquez present very thorough protocol to obtain high yield and functional mitochondria of cephalopod heart for the first time and detailed procedure for determining the respiratory parameters. A key feature that is repeated throughout is the success for reproducibility of the protocol for extracting functional mitochondria.

Authors carefully compared available methods for isolation of functional mitochondria that mainly focus on mammalian tissues, yeast, and cell cultures and certainly aware of drawbacks of each method. They also pointed out that despite marine invertebrate mitochondria studies started in early 1960s, studies mostly focused on ultrastructure of mitochondria then mitochondrial dynamics of cuttlefish hearts was an attempt on permeabilized cardiac fibers but not on isolated mitochondria.

The main strength of this paper is that it provides very first data from bioenergetics information on isolated heart mitochondria of a marine invertebrate. As such this article represents a novel study which will certainly contribute to our knowledge about mitochondrial functions and environmental adaptation of the species.

In eukaryotes, the primary hub of energy metabolism is the mitochondria, which conserve the free energy released by the biological oxidation of food-derived substrates (mostly fats and carbohydrates) in the form of a proton gradient across the inner mitochondrial membrane. In turn, this drives the synthesis of ATP, as well as other energy-requiring processes such as metabolite and ion transport, and production of heat. Some organisms (homeotherms) make use of this heat, to maintain their body temperature at an approximately constant level, whereas others (poikilotherms) tolerate a wide range of internal and intracellular temperatures. Some of the weaknesses claimed in the reasoning part of the manuscript are based on the fact that mitochondria sole function is indicated as to produce ATP. Considering the thermal tolerance of the ectotherms tightly linked to mitochondrial capability to adjust the metabolic capacity, authors should also mention the ability of mitochondria in heat production. Another possible criticism would be that based on the hypothesis postulating high temperatures could restrain female reproduction due to limited capacity of the animals’ heart to sustain oxygen flow to the body affecting energy production for the rest of the organs specifically ovaries, authors did not isolate and bioenergetically compare mitochondria from female heart.

Authors provide as main figures Respiratory Control, Respiratory State 3, Phosphorylation State, and Respiratory state 4 data but not individual “rate of oxygen consumption in each respiratory states” graphs where readers could immediately experience the functionality proof of isolated mitochondria. Instead as a supplementary information a schematic representation graph is presented. The sole purpose of the methodological paper is to provide an efficient and functional mitochondria isolated from the invertebrate heart therefore providing direct proof in the main figures would be more useful.

In the last sentence of the abstract section authors linked the amount of time required for isolation and confident and reproducibility of the result which should not be corelated, i.e, reproducible and confident results should be independent the amount of time required to isolate functional mitochondria.

The authors should revise the language to improve readability.
