# Supplementary material for: High resolution respirometry of isolated mitochondria from adult Octopus maya (Class: Cephalopoda) systemic heart
Source: PLoS One. 2022 Aug 29;17(8):e0273554. doi: 10.1371/journal.pone.0273554 (PMC9423623; doi:10.1371/journal.pone.0273554)
Supplement: Attachment — Submitted filename: Response to reviewers 0208 2022.docx [file pone.0273554.s008.docx]

Sisal, Yucatán México, August 02, 2022

Metodi D Metodiev, Ph.D.
Academic Editor
PLOS ONE

Dear Editor

Please find the response to reviewers, the manuscript's new version, and other complements of the paper “High-resolution respirometry of isolated mitochondria from adult Octopus maya (Class: Cephalopoda) systemic heart” to be considered in PLOS ONE journal.

**As can be seen, references were revised and only one citation was removed (Bradford) because this citation was included in the protocol (file: S1 step by step protocol). The RFC was changed by RCF.**

Initials of the author: CR

Grant number: 61503

Funder: Consejo Nacional de Ciencia y Tecnología

URL of Funder:

[https://conacyt.mx](https://conacyt.mx/)

Initials of the author: CR

Grant number: 204019

Funder: Dirección General de Asuntos del personal académico, universidad Nacional Autonoma de México

URL of Funder: <https://dgapa.unam.mx/>

The funders had and will not have a role in study design, data collection and analysis, decision to publish, or preparation of the manuscript.”

On behalf of the authors

Carlos Rosas

UMDI-Fac. de Ciencias, UNAM

Puerto de abrigo s/n Sisal, Yucatán

e-mail: crv@ciencias.unam.mx
